# Total Parenteral Nutrition Treatment Improves the Nutrition Status of Gynecological Cancer Patients by Improving Serum Albumin Level

**DOI:** 10.3389/fmed.2021.759387

**Published:** 2022-01-20

**Authors:** Xin Yan, Sanyuan Zhang, Junmei Jia, Jiaolin Yang, Yilai Song, Haoran Duan

**Affiliations:** ^1^Department of Drug Clinical Trial, First Hospital of Shanxi Medical University, Taiyuan, China; ^2^Department of Gynecology, First Hospital of Shanxi Medical University, Taiyuan, China; ^3^Department of Oncology, First Hospital of Shanxi Medical University, Taiyuan, China; ^4^School of Nursing, Shanxi Medical University, Taiyuan, China

**Keywords:** SG-PGA, albumin, total parenteral nutrition, gynecological cancer, malnutrition

## Abstract

**Background:**

Malnutrition is often observed in gynecological cancer patients, however its prevalence in these patients remains largely unexplored. Total parenteral nutrition (TPN) is a nutritional intervention method that has controversial treatment outcome on gynecological cancer patients. The present retrospective study is designed to evaluate the nutrition status and TPN treatment outcome on patients diagnosed with endometrial, cervical or ovarian malignant tumors.

**Methods:**

Medical records of a total of 263 patients treated at the First Hospital of Shanxi Medical University, China were included. Nutrition status was assessed by patient-generated subjective global assessment (PG-SGA). Patients were grouped based on nutrition status, cancer type or treatment strategy for clinical characteristic comparison. Multivariable logistic regression analysis was used to identify predictors for malnutrition status and hospital stay duration.

**Results:**

Presence of endometrial and cervical cancer, body weight before nutritional intervention and serum albumin level (*P* < 0.001 for all) were found to be significant predictors for malnutrition status in gynecological cancer patients. In the malnourished patients, those who were treated with TPN had significantly lower serum albumin levels before and after treatment (*P* < 0.001) and PG-SGA scores after treatment. Also, TPN treatment could significantly increase the serum albumin levels in these patients after 1 week. In addition, shorter hospitalization period was needed for TPN-treated endometrial (*P* = 0.019) and ovarian (*P* < 0.001) patients. Moreover, serum albumin levels (*P* < 0.001), use of TPN treatment (*P* = 0.025) and nutrition status (*P* = 0.010) were identified to be independent predictors for hospital stay duration.

**Conclusion:**

Our results suggest that malnutrition is a significant clinical manifestation in gynecological cancer patients who may benefit from TPN treatment for reduced hospitalization and improved serum albumin levels.

## Introduction

Malnutrition is a common situation observed in many hospitalized patients, especially in those who suffered from geriatric and malignant diseases (1). Up to 20% of the oncology patients have been considered to die from the detrimental effects of malnutrition rather than from the malignant tumor (2, 3). For surgically treated patients, malnutrition can have multiple negative impacts on various treatment stages, including increased risk of peri-operative complications (4–6), incomplete removal of the primary tumor after initial surgery (5) and increased hospitalization time (7). Patients with gynecological malignancies, especially ovarian cancer, have been shown to have higher risks to experience malnutrition (8, 9). For ovarian cancer patients, treatment strategies often involve debulking surgeries that resect parts of the gastro-intestinal tissues; therefore, these patients are theoretically more susceptible to development of malnutrition status (10–12). Previous study shows that patients with ovarian cancer were more susceptible to malnutrition at initial diagnosis (66.7%) compared with endometrial and cervical cancer (13). Another study shows that 76.1% of the patients undergoing postoperative chemotherapy for ovarian cancer were moderately or severely malnourished (14). These data suggest that the malnourishment is mainly due to the underlying pathology of ovarian cancer rather than the treatment. Malnutrition in cancer patients is a result of poor nutrient intake combined with excessive metabolic demand of the malignant tissue. This abnormally high demand was caused by an elevated production of pro-inflammatory cytokines that stimulates proteolysis and consequently leads to loss of lean tissue mass (15).

A number of different nutritional parameters have been used to assess the nutritional status in patients with gynecological cancer, in which the subjective global assessment (SGA) has been shown to be a validated nutrition assessment tool under multiple different conditions (16–18). Later, the patient-generated SGA (PG-SGA) rating and scoring systems were further developed to assess the nutrition status of patients with different types of cancer (19, 20). The check box formatted PG-SGA assessment includes changes in weight, dietary intake and functional capacity, persisted gastrointestinal symptoms, loss of subcutaneous fat, muscle wasting, ankle/sacral edema and ascites, as well as additional questions to detect nutritional symptoms and short-term weight loss (19).

Total parenteral nutrition (TPN) is an intravenous feeding method that directly delivers nutrients into the body through a vein without passing through the gastrointestinal tract. Currently, the effect of TPN treatment for malnutrition in gynecological cancer patients remains elusive. On one hand, several studies have demonstrated that TPN can improve the malnutrition status in these patients (21–23) and have life sustaining effect for terminally ill ovarian cancer patients (18). On the other hand, no improvement in survival, mitigate toxicity or tumor response rate was observed when combining TPN with adjuvant therapy (10). Current guidelines for nutritional management of gynecological cancer are mainly based on professional opinions. Therefore, extensive evidence based on clinical data from different patient populations is needed to help the doctors for decision making. In light with this, present study was designed to evaluate the nutritional status of patients with different gynecological cancers and to compare the clinical characteristics between TPN and conservative management.

## Methods

### Patients

**Figure 1:**
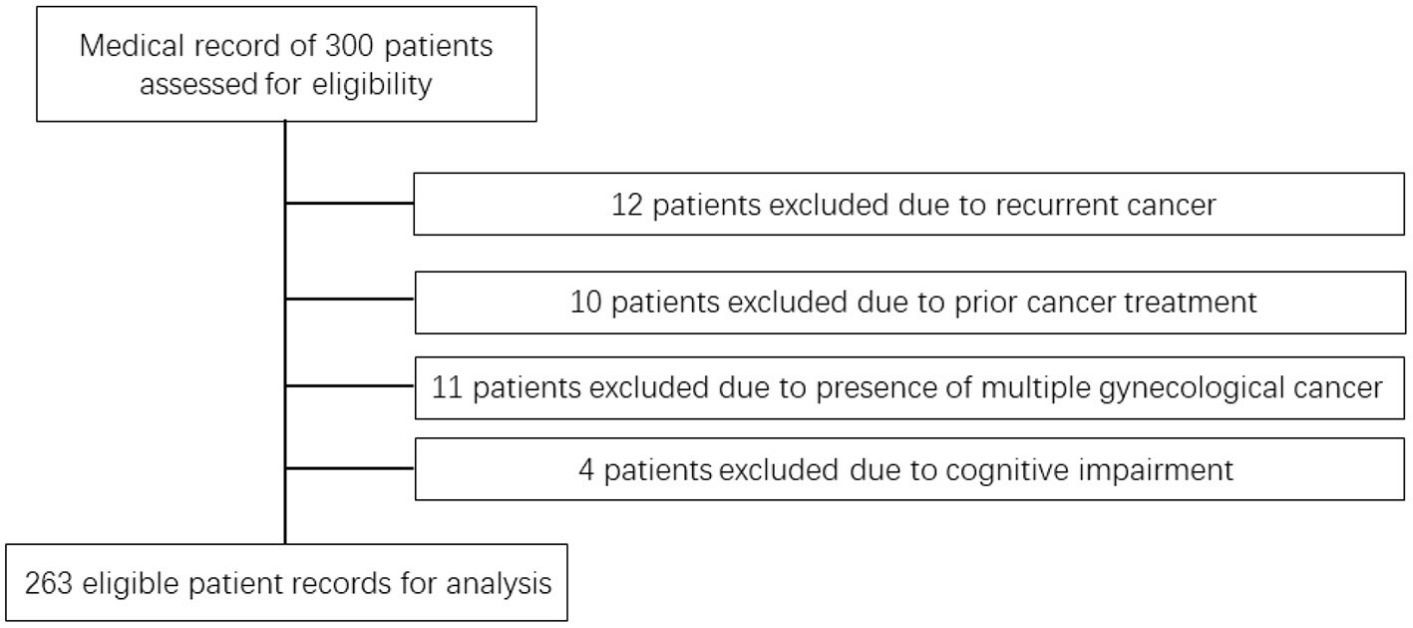
Patient selection flowchart.

Medical records of 263 participants admitted at the First Hospital of Shanxi Medical University from 2015 to 2020 were retrospectively studied. Figure 1 illustrates the patient selection flowchart. All participants were diagnosed with advanced endometrial, cervical or ovarian malignant tumor. Excluding criteria include recurrent cancer, prior treatment of other cancers within 5 years, presence of multiple gynecological cancer and cognitive impairment. All patients were examined by professional gynecological oncologists with over 10 years of experience. Tumor malignancy was determined based on histological analysis performed by experienced pathologists. Vaginal brachytherapy and/or chemotherapy were used for the treatment of endometrial cancer patients. Concurrent chemoradiotherapy was used for the treatment of cervical cancer patients. Surgery and chemotherapy were used for the treatment of ovarian cancer patients. A full medical and surgical history was recorded for each individual participant. PG-SGA assessment was performed by 2 experienced dietitians for all participants.

The present retrospective study was approved by the ethical committee of First Hospital of Shanxi Medical University (2018-28376). Guidelines outlined in the Declaration of Helsinki were followed. Written consent was obtained from all participated patients.

### Assessment of Nutrition Status

All nutritional assessments were performed 2–4 weeks before treatment using the PG-SGA rating system that allows a global assessment of patients' nutrition status on the basis of subjective and objective aspects (19). Patients' medical history used for PG-SGA includes changes in body weight, calorie intake, any symptoms that persist for at least 2 weeks and changes in functional capacity. Body weight at 1, 3 and 6 months before treatment was provided by the patients based on their own documentation record. Percentage of weight loss was calculated with the following formula: 100/past weight x (past weight – current weight). Patients were physically examined for loss of subcutaneous fat, muscle mass, ankle and/or sacral edema and ascites. According to the global rating of PG-SGA, patients were categorized into three stages, where stage A is defined as well nourished, stage B is defined as moderately malnourished, stage C is defined as severely malnourished (24).

Patients were further assessed with the scoring system of PG-SGA, where a numerical score was calculated for each patient based on the severity of each clinical feature (20). Scores of each clinical feature were added up and subsequently averaged to obtain the PG-SGA score for each patient.

### Total Parenteral Nutrition Treatment

When nutrient intake via the gastro-intestinal route failed for more than 7 consecutive days, TPN treatment was initiated (25). Otherwise, conservative nutritional management was applied under the guidance of professional dietary physicians. Patients' pre-treatment serum albumin level, body weight and PG-SGA score before and 1 week after conservative/TPN treatment, duration of hospital stay and duration of TPN treatment were documented.

### Statistics

Statistical analysis was performed using the SPSS software (version 16.0, IBM, Armonk, NY, USA). Differences in clinical variables were compared among different nutritional statuses, cancer types and treatment. Descriptive statistics were used to show the clinical characteristics. Data normal distribution was assessed by the Kolmogorov–Smirnov test. Independent *t*-test or one-way ANOVA was used to examine the differences in means for age, weight, height, BMI, serum albumin, PG-SGA score and duration of hospital stay. Categorical variables were analyzed using the χ2 test. Multivariable logistic regression analysis was performed to assess predictors of malnutrition and hospital stay duration with the adjustment of age, body mass index (BMI) and serum albumin level. A *P* < 0.05 was considered as statistically significant.

## Results

### Patient Characteristics Based on Nutrition Status

**Figure 2:**
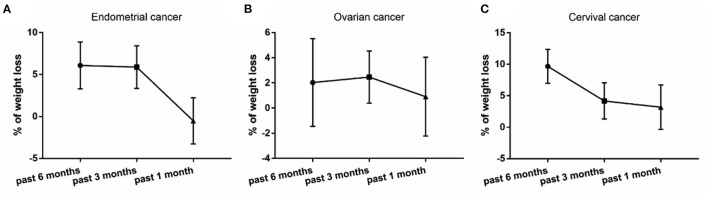
Percentage of weight loss in patients that were well-nourished **(A)**, moderately malnourished **(B)** and severely malnourished **(C)**.

We first separated the patients into 3 stages (stage A, B and C) based on the PG-SGA score. Among the 263 patients included in the present study, 55 of them were well nourished (stage A), 96 of them were moderately malnourished (stage B) and 112 of them were severely malnourished (stage C) (Table 1). No differences were observed in age or height of the 3 patient populations. Body weight at 6 months, 3 months, 1 month and right before treatment, as well as body mass index (BMI) were all found to be significantly decreased in the patients with malnourished status. When comparing the percentage of weight loss at each nutritional stage, well-nourished patients (stage A) already started to gain weight at 3 months and 1 month before treatment, as reflected by the negative percentage of weight loss at these time points (Figure 2A). For the moderately malnourished patients (stage B), the percentage of weight loss remained between 5 and 10% at all three time points (Figure 2B), indicating no weight gain during this period. For the severely malnourished patients (stage C), the percentages of weight loss were around 5% at 6 and 3 months before treatment; whereas these patients stopped weight loss at 1 month before treatment (Figure 2C). In addition, significantly lower serum albumin level was also observed in the malnourished patients (Table 1).

**Table 1:** Characteristics of patients with different nutrition status.

	**Stage A patients (*n* = 55)**	**Stage B Patients (*n* = 96)**	**Stage C Patients (*n* = 112)**	***P* value**
Age (years)	58.7 ± 8.8	60.1 ± 9.1	59.9 ± 9.0	0.638
Weight 6 months before treatment (kg)	76.4 ± 19.1	69.3 ± 11.8	58.6 ± 10.3	<0.001
Weight 3 months before treatment (kg)	71.8 ± 16.6	67.2 ± 11.5	57.7 ± 10.3	<0.001
Weight 1 month before treatment (kg)	69.3 ± 17.3	68.3 ± 9.3	54.7 ± 10.2	<0.001
Weight right before treatment (kg)	69.4 ± 17.3	61.7 ± 10.7	53.0 ± 10.2	<0.001
Height (m)	1.6 ± 0.1	1.6 ± 0.1	1.6 ± 0.1	0.137
BMI (kg/m2)	26.9 ± 6.6	23.6 ± 4.7	19.8 ± 4.4	<0.001
Serum albumin (g/L)	41.4 ± 3.6	38.2 ± 2.4	34.7 ± 2.7	<0.001
PG-SGA score	2.7 ± 1.1	14.2 ± 3.3	15.9 ± 3.4	<0.001

### Patient Characteristics Based on Cancer Type

**Figure 3:**
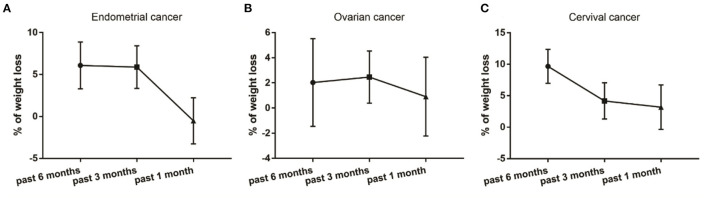
Percentage of weight loss in patients that were diagnosed with endometrial **(A)**, ovarian **(B)** and cervical **(C)** cancer.

To compare the clinical characteristics among patients with different types of gynecological cancer, we re-divided them into the endometrial, cervical and ovarian groups. We found that almost 60% of the patients with endometrial cancer suffered from severe malnourishment, whereas only about 30% of the patients with ovarian or cervical cancer experienced the same nutrition status (Table 2). Among all the studied characteristics, only body weights at 1 month and right before treatment were significantly different among the 3 groups (Table 2). Body weights of the patients with endometrial cancer dropped to the lowest level at 1 month before treatment (Figure 3A), whereas the lowest body weight levels of the patients with cervical cancer were observed at 3 months before treatment (Figure 3C). In contrast, body weights of the ovarian cancer patients were relatively steady compared to the other two types (Figure 3B).

**Table 2:** Characteristics of patients with endometrial, ovarian and cervical cancer.

	**Endometrial**	**Ovarian**	**Cervical**	***P* value**
	**(*n* = 113)**	**(*n* = 69)**	**(*n* = 81)**	
Stage A (*n*, % of total)	21 (18.6%)	16 (23.2%)	18 (22.2%)	<0.001
Stage B (*n*, % of total)	25 (22.1%)	33 (47.8%)	38 (46.9%)	<0.001
Stage C (*n*, % of total)	67 (59.3%)	20 (29.0%)	25 (30.9%)	<0.001
Age (years)	59.8 ± 9.4	60.2 ± 8.1	59.2 ± 9.2	0.785
Weight 6 months before treatment (kg)	64.2 ± 15.1	67.6 ± 15.0	67.8 ± 14.5	0.170
Weight 3 months before treatment (kg)	63.0 ± 12.5	65.9 ± 14.5	64.2 ± 14.1	0.366
Weight 1 month before treatment (kg)	59.6 ± 13.5	66.3 ± 14.4	63.9 ± 12.3	<0.001
Weight right before treatment (kg)	57.4 ± 13.1	63.4 ± 13.6	59.4 ± 13.5	<0.001
Height (m)	1.6 ± 0.1	1.6 ± 0.1	1.6 ± 0.1	0.367
BMI (kg/m2)	21.9 ± 5.2	23.8 ± 5.9	22.7 ± 6.0	0.091
Serum albumin (g/L)	36.8 ± 4.0	37.7 ± 3.7	38.0 ± 3.7	0.074
PG-SGA score	13.3 ± 5.4	12.7 ± 5.4	12.8 ± 5.3	0.659

### Prediction of Malnutrition Status

Next, we performed the multivariable logistic regression analysis adjusted for age, body weight right before treatment and serum albumin level to predict moderate and severe malnutrition status in patients with different gynecological cancer. We found that patients with endometrial and cervical, but not ovarian cancer were more prone to malnourished status (Table 3).

**Table 3:** Adjusted multivariable logistic regression analysis of factors predicting moderate and severe malnutrition in gynecological cancer patients.

	**Adjusted OR**	**95% CI**	***P* value**
Age	0.987	0.924–1.125	0.875
Body Weight before treatment	0.742	0.657–0.804	<0.001
Serum albumin	0.653	0.601–0.775	<0.001
Endometrial cancer	3.534	2.985–11.325	<0.001
Ovarian cancer	0.969	0.785–1.268	0.654
Cervical cancer	4.235	1.256–19.387	<0.001

### TPN Treatment Outcome on Malnutrition

Next, we focused on the stage B and C patients with different cancer types and divided them into two new groups based on whether they had the TPN or the conservative nutritional management. In patients with endometrial cancer, 32 of them (34.8%) received TPN treatment (Table 4). We found that serum albumin levels both before and 1 week after the nutritional treatment were significantly lower in patients who needed TPN treatment (Table 4), which was able to significantly increase the serum albumin level 1 week after treatment (*P* < 0.001). In addition, the PG-SGA score 1 week after the nutritional treatment was found to be significantly increased in the TPN-treatment patients (Table 4). However, the improvement was at the statistical significance margin when comparing the PG-SGA scores before and after the TPN treatment (*P* = 0.059). Moreover, significantly more stage C patients had to receive the TPN treatment and the TPN treatment could significantly reduce the duration of hospital stay (Table 4). In patients with cervical or ovarian cancers, 25 (39.7%) and 19 (36.5%) of them received the TPN treatment, respectively (Table 4). Similar outcome as the endometrial cancer patients was observed for the two patient groups in terms of serum albumin and PG-SGA score (Table 4). However, no significant difference was observed in nutritional stages in either patient group and the TPN treatment could only significantly reduce the duration of hospital stay in ovarian cancer patients (Table 4).

**Table 4:** Characteristics of the patients treated with either TPN or conservative management.

	**Endometrial Cancer**			**Cervical Cancer**			**Ovarian Cancer**		
	**CM (*n* = 60)**	**TPN (*n* = 32)**	***P* value**	**CM (*n* = 38)**	**TPN (*n* = 25)**	***P* value**	**CM (*n* = 33)**	**TPN (*n* = 19)**	***P* value**
Age (years,)	59.2 ± 9.0	62.3 ± 8.8	0.109	60.4 ± 9.9	57.9 ± 9.2	0.318	59.4 ± 7.9	60.6 ± 9.1	0.601
Serum albumin before treatment (g/L)	33.2 ± 5.4	20.2 ± 5.5	<0.001	32.5 ± 4.7	19.8 ± 2.9	<0.001	33.2 ± 3.9	19.3 ± 3.3	<0.001
Serum albumin after treatment (g/L)	35.3 ± 2.9	25.0 ± 3.3	<0.001	34.1 ± 2.7	25.8 ± 2.3	<0.001	34.9 ± 2.9	23.2 ± 5.1	<0.001
Body weight before treatment (kg)	65.3 ± 9.0	67.3 ± 8.3	0.301	65.4 ± 5.7	64.5 ± 5.1	0.539	63.8 ± 5.6	65.5 ± 6.3	0.348
Body weight after treatment (kg)	66.4 ± 8.9	66.9 ± 10.1	0.810	64.3 ± 4.8	61.8 ± 5.9	0.067	66.4 ± 5.7	64.4 ± 6.9	0.261
Body weight change (kg)	1.1 ± 12.9	−0.5 ± 11.3	0.578	−1.1 ± 8.0	−2.8 ± 7.7	0.425	2.6 ± 7.8	−1.0 ± 10.1	0.153
PG-SGA score before treatment	15.5 ± 3.5	15.0 ± 3.6	0.519	15.4 ± 3.1	15.7 ± 3.4	0.734	14.6 ± 3.2	14.5 ± 3.1	0.885
PG-SGA score after treatment	14.8 ± 3.1	17.0 ± 4.9	0.010	14.7 ± 3.1	16.8 ± 4.4	0.025	14.3 ± 3.1	16.3 ± 3.3	0.034
Duration of TPN (day)		3.2 ± 1.8			3.1 ± 1.3			3.2 ± 1.4	
Nutrition status (Stage B: Stage C)	36:24	10:22	0.009	22:16	11:14	0.280	10:23	9:10	0.218
Duration of hospitalization (day)	5.4 ± 3.0	3.9 ± 2.6	0.019	4.4 ± 2.5	3.5 ± 2.3	0.170	13.4 ± 3.9	9.8 ± 2.0	<0.001

*CM, Conservative management. All data were presented as mean ± standard deviation*.

Finally, we performed the multivariable logistic regression analysis adjusted for age and body weight right before treatment to identify the significant predictors for hospital stay duration in all included patients. We found that serum albumin before and 1 week after nutritional treatment, use of TPN treatment and nutrition status were significant predictors (Table 5).

**Table 5:** Multivariable logistic regression model analysis of factors predicting the hospital stay duration of the malnourished patients with endometrial, cervical or ovarian cancer.

	**Adjusted OR**	**95% CI**	***P* value**
Age	1.253	0.847–1.547	0.463
Serum albumin before treatment	4.846	2.965–6.358	<0.001
Serum albumin after treatment	3.259	2.058–4.998	<0.001
Body weight before treatment	1.142	0.947–1.298	0.753
Body weight after treatment	1.203	0.896–1.389	0.623
PG-SGA score before treatment	1.176	0.915–1.304	0.536
PG-SGA score after treatment	1.925	1.231–2.869	0.026
Use of TPN	1.958	1.324–3.059	0.025
Nutrition status	2.135	1.456–3.745	0.010

## Discussion

Not much study has been performed to document the nutrition status of patients with gynecological tumors and the standard of assessment remains controversial. To the best of our knowledge, our study is the first one to address this issue in a Chinese population, considering patients with different types of gynecological cancer, i.e. endometrial, cervical or ovarian malignant tumors. We revealed a higher tendency of malnutrition for endometrial and cervical cancer, compared to ovarian cancer. A similar previous study assessing the nutrition status in gynecological cancer patients using the scored PG-SGA has been performed on Australian population (13). However, they revealed that patients with ovarian cancer, but not endometrial or cervical cancer were prone to moderate malnourishment, which is completely opposite to our conclusion. This could be due to the different analyzed patient populations between the two studies. In addition, none of the patients were identified to be severely malnourished in the Australian study, which may also account for the observed discrepancy.

The use of TPN to treat cancer patients with poor nutritional status remains controversial. Some studies suggest that there is too little clinical benefit to warrant the nutritional intervention for such patients (10, 26). On the other hand, other studies have demonstrated significant improvement in the median survival of patients with terminal ovarian cancer who received the TPN treatment (25). In the present study, we found that TPN treatment can significantly reduce the time of hospitalization in endometrial and ovarian cancer patients as previously reported (21–23), but not in cervical cancer patients. This could be due to the relatively short hospitalization duration in the cervical cancer patients compared to the other two. Several recent studies show that oral nutrition supplements can significantly reduce the risk of malnutrition in patients undergoing chemotherapy for ovarian cancer (14) and patients following surgery for colorectal cancer (27, 28). In addition, nutrition education has been shown to have a positive effect on reducing the incidence of malnutrition in ovarian (28), head and neck (29) and breast cancer (30). In light with these data, adding these methods on top of TPN treatment might be useful for gynecological cancer patients.

In line with another previous study (31) and our recent findings (32), we found that serum albumin levels both before and after the nutritional intervention were significantly lower in the TPN treated patients. Serum albumin often reflects elevation of systemic immune response and metabolism status as a result of traumatic injury. It has been shown to be a significant predictor for operative morbidity and surgical outcome (33, 34). In addition, it has been previously used as an objective parameter to define the nutritional status in patients with gynecological cancer (13, 35). Moreover, it has also been shown to be related to surgically-induced complications, such as wound defects and septicemia in ovarian patients (5). We found similar mean albumin levels and correlation with nutrition status as these studies, indicating that it might be used as an indicator for malnutrition in gynecological cancer patients when full nutritional assessment is not possible. Successful management of hypoalbuminemia in gynecological cancer patients might be critical for their post-operative outcome. In term of future perspectives on managing the nutritional status of gynecological cancer patients, elevating serum albumin level could be a primary focus. Dietary supplementation of high-quality protein might be considered before and after surgical treatment. In addition, other serum protein indicators might be worth checking in these patients, including total protein, prealbumin, globulin and urine protein electrophoresis. Given that serum albumin is closely associated with acute and chronic inflammatory responses, levels of C-reactive protein, alpha-1 acid, glycoprotein, ferritin and ceruloplasmin could also be monitored during the course of treatment.

Weight loss and BMI have limitations on reflecting the nutrition status of gynecological patients, since the loss of lean muscle mass in some obese patients may have been masked by the excess body fat. Moreover, ascites can also affect the body weight change in gynecological cancer patients. Therefore, to assess the nutrition status of these patients, a combination of different factors should be employed. Sarcopenia, characterized by the progressive loss of skeletal muscle mass and function, is a usual clinical manifestation of gynecological malignancies. Together with skeletal muscle quality, they have been proposed to be used as predictors for postoperative complication and early mortality in patients with gynecological cancer (36). In addition, a recent review shows that sarcopenia appears to play an important role in the oncological outcomes of ovarian cancer patients (37). Therefore, interaction between TPN and sarcopenia in gynecological cancer would be an interesting direction for future studies.

There are several assessment tools for the nutrition status of cancer patients, including SGA, PG-SGA and Prognostic Nutritional Index (PNI). A previous nutritional study was conducted on 67 women with gynecological cancer using both the SGA and the PNI methods, and revealed moderate agreement between the two standards (38). Another study has proposed the scored PG-SGA as an easy-to-use nutrition assessment tool for cancer patients, which allows quick identification and prioritization of malnutrition in hospitalized patients (19). Our results further support usage of the scored PG-SGA as a tool to assess the nutrition status in patients with endometrial, cervical and ovarian cancer.

Laparoscopy, a minimally invasive approach for debulking surgery, has been widely applied to treat advanced-stage ovarian cancer (39). A recent case report successfully demonstrated a step-by-step description of the rectosigmoid mesorectal-sparing resection technique performed on a 54-year-old woman with a diagnosis of FIGO stage 3C advanced ovarian cancer (40). Future studies should be performed to specifically assess the nutritional status of gynecological cancer patients treated by these more advanced technique. In addition, prehabilitation programs and enhanced recovery after surgery (ERAS) protocols have been shown to improve the complication rate and shorten the hospital stay of gynecological cancer patients (41). A recent random trial implementing ERAS for high-complexity advanced ovarian cancer surgery suggested that ERAS should be used as a standard practice for all cytoreductive surgeries against peritoneal carcinomatosis (42). In light to this notion, the effect of ERAS on the nutritional status of these patients needs to explored in the future to better understand the mechanism of this treatment.

One limitation of the present study is its single-institutional nature. Therefore, the number and ethnicity of patients are rather limited. Future studies with larger patient quantity and variety of patient origin are needed to further verify the present findings. In addition, the heterogeneity of the included patient population might cause certain basal variations in their body weight, which may complicate the assessment of nutrition status.

## Conclusion

In summary, we found that women with endometrial and cervical cancer were more prone to suffer malnutrition, compared to ovarian cancer patients. In addition, we show that lower serum albumin levels, use of TPN treatment and nutrition status were all closely related to the length of hospital stay of gynecological patients. Our study further supports the scored PG-SGA as a useful tool to detect the nutrition status in gynecological cancer patients and suggest that TPN should be considered as a positive treatment method in these patients.

## Data Availability Statement

The original contributions presented in the study are included in the article/supplementary material, further inquiries can be directed to the corresponding author.

## Ethics Statement

The studies involving human participants were reviewed and approved by Ethical Committee of First Hospital of Shanxi Medical University. The patients/participants provided their written informed consent to participate in this study.

## Author Contributions

XY and SZ designed and performed the study. XY wrote the manuscript. JJ, JY, YS, and HD helped with data analysis. All authors contributed to the article and approved the submitted version.

## Conflict of Interest

The authors declare that the research was conducted in the absence of any commercial or financial relationships that could be construed as a potential conflict of interest.

## Publisher's Note

All claims expressed in this article are solely those of the authors and do not necessarily represent those of their affiliated organizations, or those of the publisher, the editors and the reviewers. Any product that may be evaluated in this article, or claim that may be made by its manufacturer, is not guaranteed or endorsed by the publisher.

## Abbreviations

SGA: subjective global assessment
PG-SGA: patient-generated SGA
BMI: body mass index
TPN: total parenteral nutrition
PNI: Prognostic Nutritional Index.
